# Examining Attitudes Related to Trauma-Informed Care Among School Professionals: A Baseline District-Wide Organizational Assessment

**DOI:** 10.1007/s40653-026-00955-7

**Published:** 2026-08-04

**Authors:** Suzanne M. Ackers, Jessica Grace

**Affiliations:** 1 Department of Nursing, Texas Christian University, Fort Worth, USA; 2 Department of Social Work, University of North Texas, Denton, USA

**Keywords:** Trauma-informed care, Trauma-informed approach, School, School professional, Mental health, Youth, Attitudes, ARTIC, SAMHSA

## Abstract

Children’s exposure to trauma can significantly affect their learning and behavior in school. Trauma-informed systems, including schools, can support youth who have experienced adverse childhood experiences by creating safe environments that avoid retraumatization and restore academic, health, and developmental trajectories. The purpose of this study was to obtain a baseline organizational assessment of a large Texas school district’s readiness to implement trauma-informed care (TIC). The study followed the first step in the Substance Abuse and Mental Health Services Administration’s guidelines for implementing TIC: determining organizational readiness. An exploratory cross-sectional design was employed, using the Attitudes Related to Trauma-Informed Care instrument to measure the district’s readiness by assessing staff attitudes toward TIC, their confidence in implementing TIC, and perceived organizational support for adoption among school professionals. Descriptive statistics were examined, and inferential testing included omnibus one-way ANOVA, paired *t* tests, and post hoc Bonferroni pairwise comparisons. The final sample consisted of 211 participants (*N* = 211), who included teachers, counselors, administrators, and nurses. Of these, 56% had reported prior TIC training (*n* = 119), and 44% had not (*n* = 92). Results showed that the On-the-Job Behavior subscale scored highest, with respondents endorsing empathy-focused staff behavior over control-focused behavior. The Personal Support subscale scored higher than the System Support subscale for staff who reported prior TIC training, indicating confidence and support for implementing TIC. Significant group differences between trained and untrained participants suggest that trauma-informed training in this sample was effective. Continued TIC training, as mandated by the state, shows promising results in helping school professionals develop readiness to understand trauma and its contribution to behavioral issues in U.S. schools. Future research with the same district will employ a qualitative design to identify facilitators and barriers to implementing TIC in school settings. Developing a trauma-informed school environment and implementing TIC can contribute meaningfully to the growing body of implementation science and the evidence base in trauma research.

Children’s exposure to trauma can affect their learning and behavior in school. Schools are a critical system of support for students who have experienced trauma, making school professionals an integral part of helping to reduce the effects of trauma by becoming trauma-informed (American Psychological Association, 2025; National Child Traumatic Stress Network, 2017. In the United States (U.S.), 61% of adults report exposure to at least one adverse childhood experience (ACE), three out of four high school students report experiencing at least one ACE, and over two-thirds of children report experiencing at least one traumatic event by age 16 (Bhushan 2020) Centers for Disease Control and Prevention [CDC], 2021; (Kilpatrick et al. 2013) Substance Abuse and Mental Health Services Administration [SAMHSA], 2026). Additionally, research suggests that adverse social determinants of health (SDOH), such as poverty, food insecurity, housing instability, and exposure to community violence, can increase children’s risk of trauma exposure and ACEs, which may negatively affect health and well-being across the lifespan (Baker et al. 2016) National Council of State Education Associations [NCSEA], 2019). These statistics underscore the need for trauma-informed schools that are equipped to support students affected by these adversities. Adverse childhood experiences are potentially traumatic events, including abuse and neglect, that occur before age 18 and directly affect long-term adolescent and adult health (CDC, 2021; (Felitti et al. 1998). A trauma-informed care approach includes the realization of how ACEs affect health and a response that avoids retraumatization (Goddard 2021). School districts have access to large numbers of youth and, therefore, are uniquely positioned to recognize childhood trauma and intervene early, before lifelong physical and psychological health consequences associated with trauma ensue, by implementing a district-wide trauma-informed approach.

Many ACEs are preventable; however, schools cannot necessarily prevent them from occurring because many occur outside the school environment. Rather, by providing safe and supportive environments, trauma-informed schools can help mitigate the negative consequences of ACEs from escalating and interfering with students’ health, development, and academic success (CDC, 2024b). To this point, although schools cannot prevent all ACEs, estimates show that efforts to reduce risk factors, childhood adversity, and strengthen protective factors may contribute to lower rates of suicide attempts among high school students, depression, prescription pain medication misuse, feelings of sadness/hopelessness, and the later development of heart disease (Bhushan 2020; Merrick et al. 2019). Moreover, youth who experience ACEs are more likely to exhibit negative behaviors at school and to develop risky health behaviors over their lifetime (CDC, 2021). Trauma-informed systems, including schools, have the potential to support youth who have experienced an ACE(s) by creating an environment that fosters safety, prevents retraumatization, and promotes the restoration of developmental trajectories (Baker et al. 2016) SAMHSA, 2023).

The Substance Abuse and Mental Health Services Administration (SAMHSA, 2023) proposes that systems and organizations that implement trauma-informed approaches create a safer environment for both their staff and the individuals they serve. Consistent with SAMHSA’s (2023) recommendation to implement trauma-informed care through organization-wide transformation, understanding trauma-informed attitudes among school employees may provide insight into workforce characteristics that support implementation. Although trauma-informed attitudes are not synonymous with organizational readiness, they are an important component of readiness and can help identify organizational strengths and areas that require additional support to facilitate sustainable, system-wide implementation of trauma-informed care. Likewise, examining differences in staff attitudes can provide insight into organizational readiness for TIC implementation to support trauma-affected students. Recognizing the importance of assessing workforce attitudes as part of organizational readiness and planning, the SAMHSA identified the Attitudes Related to Trauma-Informed Care (ARTIC) scale as a recommended measure of trauma-informed attitudes to inform readiness to implement trauma-informed care (SAMHSA, 2023, p. 16).

Guided by SAMHSA’s framework for implementing a trauma-informed approach, this study used the Social Cognitive Theory (SCT) to operationalize trauma-informed attitude variables and employed the ARTIC scale to assess attitudes that may provide insight into organizational readiness for trauma-informed implementation. In this study, the terms trauma-informed care (TIC) and trauma-informed approach (TIA) will be used interchangeably, as defined by the SAMHSA (2023), the guide used for the study. According to the SAMHSA (2023), a TIC or a TIA is a program, organization, or system that is trauma-informed and realizes the widespread impact of trauma and how to recognize signs and symptoms of trauma in all individuals that are part of the system and responds by fully integrating knowledge about trauma into policies, procedures, and practices, all of which seek to resist retraumatization.

## Background

Whether it is hearing about the grim reality of another U.S. school shooting or the daily toll of violence, poverty, abuse, and addiction occurring in U.S. communities today, trauma seems to be hidden in plain sight. The NEA and the National Council of State Education Associations (NCSEA) have recognized the need to address trauma in schools, given its high prevalence and the risk of poor academic and health outcomes (CDC, 2021; NCSEA, 2019; NEA, 2025). Additionally, in October 2024, the National Institute of Nursing Research (NINR) announced plans to include school health as its second strategic imperative, emphasizing the importance of school systems in the current and future health and well-being of the student population (NINR, 2024). Likewise, the SAMHSA (2023) has recognized the critical role schools play in addressing trauma among student youth and recommends that schools implement a TIA. In their landmark publication discussing the concept of trauma, the SAMHSA (2014) influenced the expansion of provisions to address trauma outside of behavioral health and health care, including anyone involved in organization-level change, such as practitioners, state and local officials, policymakers, federal and non-federal funders, peers, and family members. Recently, to follow up on their landmark 2014 publication, the SAMHSA (2023) has released a comprehensive practical guide for implementing a TIA listing schools as a primary starting point. For example, the end of the guide contains three case studies that illustrate a TIA in action across three different systems and organizations, one of which is Fall-Hamilton Elementary School in Nashville, TN, which began its transformation toward implementing a TIA in 2015 (SAMHSA, 2023, p. 34).

The SAMHSA (2023) guide outlines three comprehensive steps for successfully implementing a TIA within systems and organizations. The first step in SAMHSA’s guide is an organizational assessment, a baseline to determine readiness to begin implementing TIC among students, youth, and employees alike, since trauma histories can affect everyone. The second step involves implementing a TIA, and the third is evaluation and sustainability. All steps in the guide include abundant data and are rich with resources, instruments, and tips to facilitate TIA organizational success. This study focuses on collecting and analyzing data for the first step of the guide: a baseline organizational assessment.

Despite increasing interest in trauma-informed schools, little is known about how trauma-informed attitudes vary among teachers, school nurses, counselors, and other school employees within large school districts or how differences may influence organizational readiness for system-wide implementation. Existing research has focused largely on individual educators, primarily teachers, or training outcomes, leaving a gap in understanding the role-specific attitudes that may facilitate or hinder trauma-informed transformation at the organizational level (Baker et al. 2016; Blanton et al. 2022; Liang et al. 2020; Minne and Gorelik 2021). Consequently, there remains a need for district-level investigations that assess trauma-informed attitudes across diverse employee groups to better understand organizational readiness and guide implementation efforts.

Texas school districts have been required to implement TIC policies since 2019 (Texas Education Code § 38.036, 2019); however, recent state legislation requires them to phase in mandatory mental health and trauma training for school employees through the 2028–2029 school year (Texas Education Code § 38.351, 2024). Beginning in 2024, the Texas Education Agency (TEA) requires each school district employee in the state who regularly interacts with students to complete an evidence-based mental health training program, and districts have five years to meet the requirement (TEA House Bill 3, 2023). In Texas, the Youth Mental Health First Aid (YMHFA) training meets the legislation requirement for mental health training (TEA House Bill 3, 2023) and is being implemented in the sample district. The YMHFA training program is supported and administered by the National Council for Mental Wellbeing 2026 and is a structured, evidence-based program designed to teach adults how to help youth experiencing mental health challenges or in crisis, and it includes TIC concepts (National Council for Mental Wellbeing, 2026. The Youth Mental Health First Aid training introduces key TIC concepts, such as non-judgmental communication, recognizing trauma exposure as a risk factor to youth mental health challenges and distress, and supportive and empathetic strategies for responding. The YMHFA training uses the terms *trauma*,* adverse experiences*, and *post-traumatic stress disorder* as part of the training; however, it is not considered a trauma-informed care model or framework in the same way as the SAMHSA’s TIC framework. Specifically, YMHFA training and SAMHSA’s TIC framework share many commonalities in recognizing and responding to trauma; however, YMHFA is skills-based, whereas the SAMHSA’s TIC framework is a system-wide approach that guides organizational practices and culture (National Council for Mental Wellbeing, 2026; SAMHSA, 2023). The YMHFA training is beneficial for school staff, nurses, youth group leaders, parents, coaches, and others who interact with youth, as it provides a foundation for understanding trauma and adopting TIC principles and strategies in organizational and school settings. Participation in the training is expected to improve mental health literacy, increase confidence in responding to mental health challenges, and promote the use of trauma-sensitive communication and response strategies consistent with trauma-informed principles (National Council for Mental Wellbeing, 2026.

The school district in this study has begun implementing the YMHFA for some of its employees, while others have not yet received it. Specifically, for the 2024/25 academic year, the district has provided the YMHFA training to all secondary-level (i.e., 6th −12th grade) teachers, administrators, and paraprofessionals. Next academic year (i.e., 2025/26), training will be provided to the same groups at the elementary level (i.e., Pre-K- 5th grade). Ultimately, all staff in the district will receive the training, no matter their part-time or full-time status.

Recommendations from state and national organizations that support school health and safety underscore the need for schools to implement a system-wide, trauma-informed approach consistent with SAMHSA’s (2023) guidance (CDC, 2021; National Association of School Nurses, 2021; National Child Traumatic Stress Network, 2017; National Education Association, 2025; Texas Education Code § 38.351, 2024). This study is exploratory, and its findings contribute to the current state of the science on the use of TIAs in a district-wide, organizational school setting. While at the same time providing the sample district with valuable insight about (a) their employees’ current TIC attitudes and competencies; (b) areas to address related to a trauma-informed approach; and (c) a benchmark or baseline for measuring progress toward implementation, the second step in SAMHSA’s guide.

## Study Purpose and Research Questions

The purpose of this study was to conduct a baseline district-wide, organizational assessment of a Texas school district’s readiness to implement a TIA, following the SAMHSA (2023) guide for the successful implementation of TIAs in organizations. In this study, organizational readiness refers to the extent to which school employees have favorable attitudes towards TIC, confidence in applying TIAs, and perceived organizational support for implementation. The SCT (Bandura 1977)informed the operationalization of the study variables by providing a framework for conceptualizing and measuring trauma-informed attitudes, using the ARTIC scale (Baker et al. 2021). The ARTIC scale was selected for the study because SAMHSA (2023, p. 16) identified it as a recommended measure for assessing attitudes toward TIC and informing organizational readiness for implementation. The ARTIC examined factors associated with school professionals’ attitudes toward TIC, their confidence in implementing a TIA, and organizational support for adopting TIC within the school environment. Ultimately, this study addresses the National Education Association’s^2025^ deliberate efforts to transform U.S. school districts into trauma-informed environments for students and employees.

**Fig. 1:**
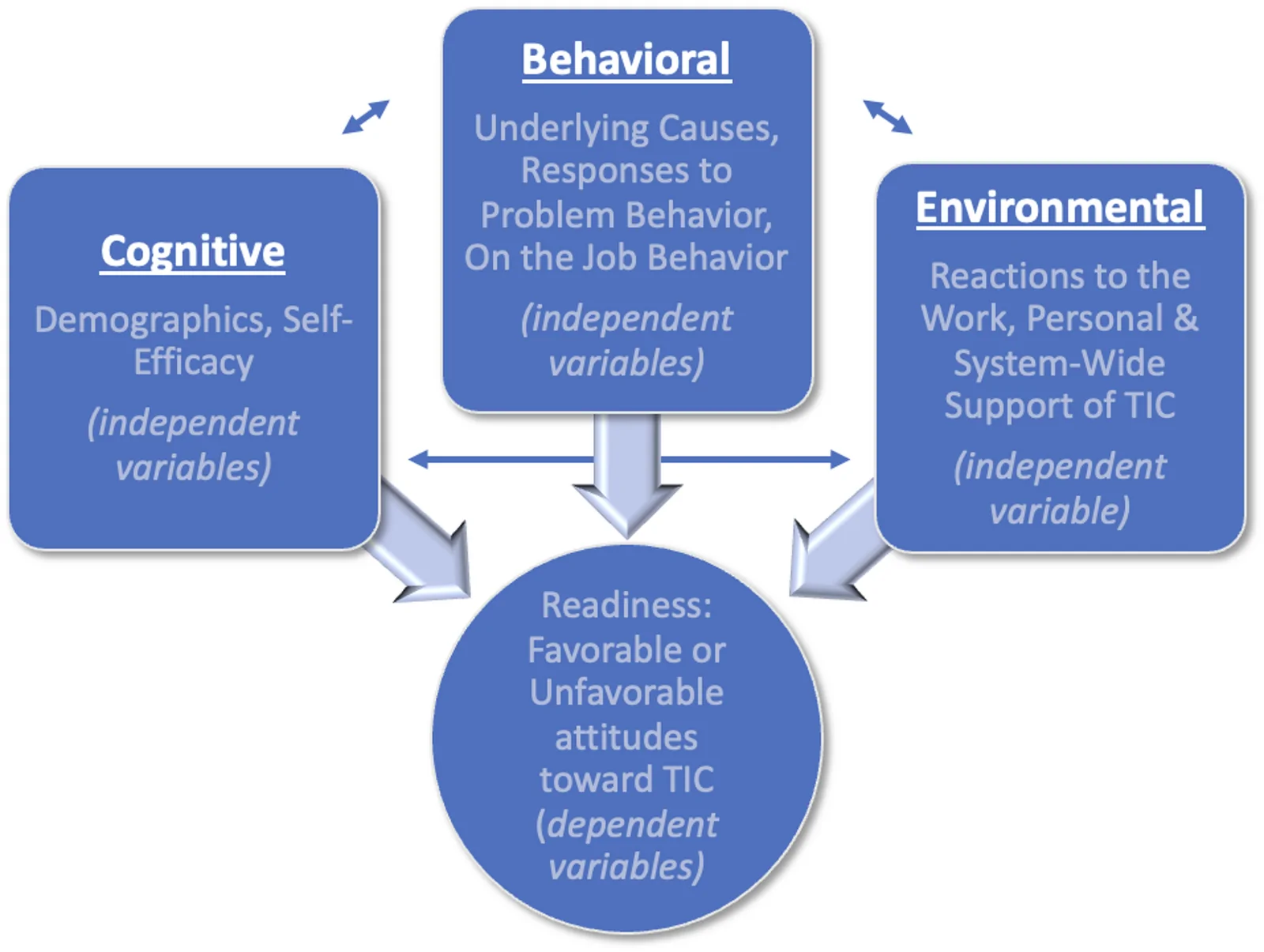
A conceptual model of the hypothesized factors that influence school professionals' attitudes related to TIC operationalized within the ARTIC 45 subscales, organized according to the measurement of the independent and dependent study variables (Bandura, 1977)

This study addresses three research questions. The study variables were operationalized within the main constructs of the Social Cognitive Theory (i.e., cognitive, behavioral, and environmental) and organized according to the ARTIC subscales and the measurement of independent and dependent variables (Baker et al. 2021; Bandura 1977). The Social Cognitive Theory (SCT) provides a useful framework for understanding factors that may influence the adoption of trauma-informed practices among school employees. The SCT (Bandura 1977) posits that behavior is shaped by the reciprocal interaction among personal factors (i.e., cognitive), behavioral experiences (i.e., behavioral), and environmental influences (i.e., environmental). Constructs reflected in the ARTIC, such as beliefs about the causes of student behaviors, confidence in responding to trauma-affected students, and perceptions of workplace practices, align with SCT concepts. Figure 1 illustrates the primary constructs of the Social Cognitive Theory and their operationalization within the ARTIC 45, organized according to independent and dependent variables. Research questions examined were:

1. What are TIC attitudes among school professionals?
2. Are there differences in attitudes related to TIC between school professionals who have received TIC training versus those who have not?
3. Do attitudes related to TIC differ across school professional roles?

## Methodology

### Study Design, Ethical Approval, and Data Collection

This study employs an exploratory cross-sectional design, utilizing an anonymous electronic survey sent to employees in a Texas school district. The survey included the ARTIC instrument and a demographic questionnaire, both created in Qualtrics (Qualtrics, 2022. This study was approved and granted exemption status from the Institutional Review Board of the principal investigator’s institution (IRB #2024/391). The principal investigator (PI) for the study met with the school district’s superintendent, and written consent was obtained to conduct the study. A district liaison was assigned to collaborate with the PI. An invitation to participate was electronically sent via email to 2,468 district employees, and a reminder email was sent one week later. Data were collected in February and March 2025.

### Sample

The district from which this study sample was drawn is considered a large suburban district in Texas, with an enrollment of over 23,000 students and employing 3,800 staff (TEA, 2020). Of the 2,468 emails initially sent, 348 responses were received, resulting in a 14.1% initial response rate. Time was spent cleaning the data, including deleting incomplete responses and reverse-coding. After data cleaning, 211 respondents were included in the study, yielding an overall response rate of 8.5%. Of the 211 respondents, 119 met the criteria for designation as a TIC-trained participant, while the remaining 92 did not.

The sample represented a range of roles across elementary, middle, and high school settings, allowing for examination of attitudes across disciplines and grade levels. Participants also self-reported prior exposure to TIC-related training; however, the study did not differentiate among specific types of training (e.g., YMHFA versus other TIC-related training). Since the ARTIC was designed to be administered to those who have not been introduced to TIC, as well as those with some TIC knowledge, the inclusion criteria aimed to capture both groups, as measuring TIC differences among participants addresses the second research question. Inclusion criteria were current employees (a) who work full-time or part-time; (b) who are at least 18 years of age; (c) who work directly on a school campus (e.g., elementary, middle, or high school); (d) who have some regular face-to-face contact with students. A specific criterion for administering the ARTIC survey is that participants must have some regular face-to-face contact with student youth. Those excluded from the study are district-level employees who work exclusively at the Central Administration Building and do not have regular face-to-face contact with students.

### Recruitment Process and Informed Consent

A purposive, random sample of current employees in the school district was recruited. The district sent the anonymous electronic survey to all employees who met the inclusion criteria on a specific Professional Development (PD) day in February 2025. The goal was to send the survey link on a PD day to boost the response rate among employees interested in participating. The survey remained open for two weeks after the PD day to capture any participants who missed it or were absent. All recruitment emails sent to prospective participants were sent through the district. The recruitment process included three phases of emails distributed to those who met the inclusion criteria using the following ordered strategy:

1. An initial email was sent to notify prospective participants about the study and its availability, starting on the PD day.
2. A recruitment email inviting prospective participants containing the link to the informed consent and survey was sent on the PD day.
3. A one-time reminder email.

This study was conducted in a manner that promotes confidentiality, anonymity, autonomy, well-being, and participants’ rights. The consent form explains that participation is voluntary and that participants have the right to refuse to participate or to stop answering questions at any time without repercussions. Informed consent to participate was included on the first page of the Qualtrics survey, and by clicking the link to participate at the bottom of the consent form, the individual agreed to participate. Similarly, Qualtrics responses are configured to anonymize, ensuring that the study cannot link individual responses to participant identities or IP addresses, thereby maintaining anonymity and confidentiality.

### Measures

All variables in the study were measured using an existing instrument. The Traumatic Stress Institute of Klingberg Family Centers developed the ARTIC survey. This institute promotes excellence in trauma-informed services not only within the Klingberg Center but nationally and internationally to organizations that treat clients with psychological trauma histories (Traumatic Stress Institute, 2024. Before the development of the ARTIC, there was no objective way to determine the extent to which an individual or organization practiced TIC. The many variations in definitions of TIC were vague and offered little guidance on how to operationalize the concept. The creators of the ARTIC sought to develop a measure that would reflect and synthesize current theoretical and empirical knowledge related to TIC, assess service providers’ attitudes relevant to TIC directly and specifically, and be relatively inexpensive to administer and score by diverse institutions such as schools, human service agencies, and health care organizations (Baker et al. 2016) p. 64).

In total, there are six versions of the ARTIC scale available publicly: three for human service organizations and 3 for education settings. The only difference is in surface-level language only (i.e., “student” vs. “client”). Of the three ARTIC versions available for educational settings, ARTIC 45 is used in this study. The ARTIC 45 contains 45 items and seven subscales (5 core [7 items each]) and two supplementary (5 items each), and is used in organizations that have already begun to implement trauma-informed care. Subscales are (1) underlying causes of problem behavior and symptoms; (2) responses to problem behavior and symptoms; (3) on-the-job behavior; (4) self-efficacy at work; (5) reactions to the work; (6) personal support for TIC; and (7) system-wide support for TIC. Items 1–35 on the ARTIC are designed for respondents, no matter their level of TIC knowledge. Whereas the last ten items (36–45) are designed for respondents with some knowledge and familiarity with TIC (Baker et al. 2016). The first 35 items are based on a 7-point Likert scale, asking respondents to select the circle along the dimension between the two options that best represents their personal belief at work over the past two months. Likewise, the last ten items also ask respondents to select a circle along the dimension that best represents their belief; however, these items are scored on an 8-point Likert scale, with option eight being “N/A.” Table1 includes the ARTIC 45 subscales and the meaning of each subscale.

**Table 1:** ARTIC 45 subscales and subscale meaning

Five Core and Two Supplemental	Subscale Meaning
1. Underlying Causes (core)	Emphasizes behavior and symptoms as adaptations & malleable vs. intentional & fixed
2. Responses (core)	Emphasizes relationships, flexibility, kindness, & safety as agent of change vs. rules, consequences and accountability
3. On-the-job behavior (core)	Endorses empathy-focused staff behavior vs. control-focused
4. Self-efficacy at work (core)	Endorses feeling able to meet the demands of working with a traumatized population vs. feeling unable to
5. Reactions to the work (core).	Endorses appreciating the effects of vicarious traumatization & coping by seeking support vs. minimizing the effects and ignoring it
6. Personal support for TIC (supplemental)	Endorses being supportive of and confident about implementation of TIC vs. concerns about implementing TIC
7. System-wide support for TIC (supplemental)	Endorses feeling system-wide support for TIC vs. not feeling supported by colleagues, supervisors, and administration to implement TIC

*vs* versus. (Baker et al. 2021)

The demographic section of the survey was developed based on recommendations from the ARTIC creators and feedback from the PI’s district liaison, who provided input on the wording of demographic items related to school roles that district employee respondents would recognize. The demographic section is located at the end of the survey, after the ARTIC items. Specifically, after respondents answer the first 35 items, they will be asked the following yes/no question: *Have you received prior training and/or education in Trauma-Informed Care?* If the respondent answers *No*, the survey will direct them to the demographic section; whereas, if they respond *Yes*, they will be directed to the last ten items (items 36–45) on the ARTIC and then to the demographic section.

Scoring for the ARTIC is based on the sum for each subscale; higher scores indicate a favorable attitude toward TIC, and lower scores indicate an unfavorable attitude. The ARTIC has undergone psychometric evaluation using confirmatory factor analysis, Pearson’s product-moment correlations, and Cronbach’s alpha, all of which support the instrument’s reliability and validity as a potentially valuable clinical research tool (Baker et al. 2016, 2021).

## Data Analysis

Data were analyzed using IBM SPSS Statistics version 29 (IBM Corporation, 2024. The PI consulted with a statistician to assist with data cleaning and analysis for both descriptive and inferential statistics. Descriptive statistics were analyzed using frequencies and means. Inferential measurement utilized omnibus one-way ANOVA, paired *t* tests, and post hoc Bonferroni pairwise comparisons.

Participants were designated as having received prior TIC training if they met two predetermined criteria. First, participants must have self-reported receiving prior TIC training by responding *yes* to the question, “*Have you received prior training and/or education in Trauma-Informed Care?”*The second criterion was based on the ARTIC scoring rules for completing the two supplemental subscales, which were answered only by those who indicated having prior TIC training. These supplemental subscales can be calculated for a given respondent if they answered at least three out of the five items from the two supplemental subscales. If this minimum has not been met, scores for these subscales are considered invalid (Baker et al. 2021). Nine participants in this study indicated having had prior TIC training but did not have valid scores from the supplemental subscales, so they were not included in the TIC-trained group (*n* = 9).

All participants in the study completed the ARTIC 35 items (*N* = 211), while those in the TIC training group completed the ARTIC 45’s additional 10 items (*n* = 119). Each participant’s score was averaged across the number of questions for an overall ARTIC 35 score and averaged across specific items designed to measure the five subscales for the ARTIC 35: Underlying Causes, Responses, On-the-Job Behavior, Self-Efficacy, and Reactions. For those who completed the additional 10 items, scores were also averaged across all items to obtain an overall ARTIC 45 score and across the specific items designed to measure the ARTIC 45 supplemental subscales for Personal Support and System Support. Because all ARTIC items were scored on a scale from 1 to 7, overall average and subscale scores also ranged from 1 to 7.

To adjust for issues with multiplicity in the study, a Bonferroni-adjusted level of significance of 0.003 was used for research questions one and two, calculated by dividing a 0.05 level of significance by the 17 total *t* tests. The third research question did not apply a Bonferroni adjustment to the significance level for the omnibus one-way ANOVAs, but pairwise comparisons were conducted using Bonferroni post hoc tests.

## Results

The final sample comprised 211 participants who fully completed the ARTIC instrument. Of these, 119 were identified as having prior TIC training based on the inclusion criteria, and 92 were identified as not having received prior TIC training (*N* = 211). The results section is presented below, beginning with the demographic section, followed by the three research questions in order.

### Demographics

Participants had an average age of 44.53 years (*SD* = 8.719, *n* = 206), ranging from 23 to 68 years. The average tenure in the educational system was 15.395 years (*SD* = 8.439, *n* = 210), with an average of 7.852 years in their current district (*SD* = 6.265, *n* = 210). Tenure in the educational system ranged from 0.5 to 40 years, while tenure in the district was capped at 30 years. Most of the participants were White (74.9%, *n* = 158), followed by Hispanic or Latino (10.9%, *n* = 23), Black (4.7%, *n* = 10), multiracial (1.9%, *n* = 4), Asian (0.5%, *n* = 1), and those who preferred not to answer (7.1%, *n* = 15). The majority had a Master’s degree (47.9%, *n* = 101) or Bachelor’s degree (43.6%, *n* = 92), with only 4.3% having lower than a Bachelor’s degree (*n* = 9) and 3.8% having higher than a Master’s degree (*n* = 8).

Most participants were full-time employees (98.6%, *n* = 208) who were not coaches (87.7%, *n* = 185), and just under half had experience working in multiple roles (46.0%, *n* = 97). The majority were teachers (68.7%, *n* = 145), followed by counselors or nurses (18.0%, *n* = 38), campus administrators (5.7%, *n* = 12), paraprofessionals (5.2%, *n* = 11), and special aides/trainers/specialists (2.4%, *n* = 5). Notably, demographic results indicate that only one participant identified as a school nurse; therefore, counselors and nurses were combined into a single category for descriptive analysis (i.e., counselors or nurses). Almost half (47.9%, *n* = 101) worked primarily on elementary school campuses, followed by high school campuses (35.1%, *n* = 74) and middle school campuses (16.6%, *n* = 35).

Participants who met the criteria for having TIC training represented 56.4% of the sample (*n* = 119) versus 43.6% who were not in the TIC-trained group (*n* = 92). Participant demographics differed between these two groups for some categories within race/ethnicity, highest level of education, primary campus, and primary role. Independent *z* tests were run to determine if any of these categories had significantly different proportions between trained and untrained participants. With race/ethnicity, results indicated the trained group had a significantly lower proportion (*z* = −2.296, *p* =.022) of multiracial participants (0.0%, *n* = 0) than the untrained group (4.3%, *n* = 4). With highest level of education, the trained group had a significantly lower proportion (*z* = −3.328, *p* <.001) of participants with a bachelor’s degree (33.6%, *n* = 40) than the untrained group (56.5%, *n* = 52), corresponding to a significantly higher proportion (*z* = 3.345, *p* <.001) of participants in the trained group with a master’s degree (58.0%, *n* = 69) than the untrained group (34.8%, *n* = 32). With primary campus, there was a significantly higher proportion (*z* = 2.160, *p* =.031) of participants in the trained group working primarily at a high school campus (41.5%, *n* = 49) than the untrained group (27.2%, *n* = 25). Finally, with primary role, there was a significantly lower proportion (*z* = 3.345, *p* <.001) of participants working as teachers in the trained group (60.5%, *n* = 72) than the untrained group (79.3%, *n* = 73); significantly higher proportion (*z* = 3.457, *p* <.001) of participants in the trained group working as a counselor or nurse (26.1%, *n* = 31) than the untrained group (7.6%, *n* = 7); and significantly higher proportion (*z* = 2.001, *p* =.045) of participants in the trained group working as a paraprofessional (2.5%, *n* = 3) than the untrained group (8.7%, *n* = 8). No other differences in demographics between the two groups were significant. A summary of these demographics for all participants, along with a breakdown for TIC-trained participants versus untrained participants, can be found in Table 2.

**Table 2:** Summary of selected demographics

Variable	Overall		Trained		Untrained	
	%	*n*	%	*n*	%	*n*
Race/Ethnicity						
White	74.9	158	74.8	89	75.0	69
Hispanic or Latino	10.9	23	13.4	16	7.6	7
Black or African American	4.7	10	2.5	3	7.6	7
Multiracial	1.9	4	0.0	0	4.3	4
Asian	0.5	1	0.8	1	0.0	0
Preferred Not to Answer	7.1	15	8.4	10	5.4	5
Highest Level of Education						
Lower than a Bachelor’s Degree	4.3	9	3.4	4	5.4	5
Bachelor’s Degree	43.6	92	33.6	40	56.5	52
Master’s Degree	47.9	101	58.0	69	34.8	32
Higher Than Master’s Degree	3.8	8	4.2	5	3.3	3
Preferred Not to Answer	0.5	1	0.8	1	0.0	0
Primary Campus						
Elementary School Campus	48.1	101	42.4	50	55.4	51
Middle School Campus	16.7	35	16.1	19	17.4	16
High School Campus	35.2	74	41.5	49	27.2	25
Primary Role						
Teacher	68.7	145	60.5	72	79.3	73
Counselor or Nurse	18.0	38	26.1	31	7.6	7
Campus Administrator	5.7	12	7.6	9	3.3	3
Paraprofessional	5.2	11	2.5	3	8.7	8
Special Aide/Trainer/Specialist	2.4	5	3.4	4	1.1	1

Overall *N* = 211; Trained *N* = 119; Untrained *N* = 92, except in primary role where Trained had *N* = 118 due to one non-response

### Research Question One: School Professionals’ TIC Attitudes

The first research question was designed to examine the cognitive, behavioral, and environmental attitudes related to TIC among school professionals. The mean overall ARTIC 35 score was 5.149 (*SD* = 0.665), while the mean overall ARTIC 45 score was 5.081 (*SD* = 5.081). The ARTIC 35 subscale for On-the-Job Behavior had the highest score (*M* = 5.416, *SD* = 0.690), followed by Self-Efficacy (*M* = 5.328, *SD* = 0.952), Reactions (*M* = 5.200, *SD* = 0.816), Responses (*M* = 5.014, *SD* = 0.902), and Underlying Causes (*M* = 4.787, *SD* = 0.793). The ARTIC 45 subscale for Personal Support had a higher score (*M* = 5.045, *SD* = 1.050) than System Support (*M* = 3.498, *SD* = 0.700).

A paired *t* test was used to determine if the differences in mean scores across the five ARTIC 35 subscales and between the two ARTIC 45 subscales were significant. Results indicated that the mean score for On-the-Job Behavior was significantly higher than the mean score for Underlying Causes (*t*(210) = 12.597, *p* <.001, Cohen’s *d* = 0.867), Responses (*t*(210) = 8.859, *p* <.001, Cohen’s *d* = 0.591), and Reactions (*t*(210) = 4.551, *p* <.001, Cohen’s *d* = 0.313). The mean score for Self-Efficacy was significantly higher than the mean score for Underlying Causes (*t*(210) = 8.736, *p* <.001, Cohen’s *d* = 0.601) and Responses (*t*(210) = 4.863, *p* <.001, Cohen’s *d* = 0.335). The mean score for Reactions was significantly higher than the mean score for Underlying Causes (*t*(210) = 7.457, *p* <.001, Cohen’s *d* = 0.513) and Responses (*t*(210) = 3.135, *p* <.001, Cohen’s *d* = 0.216). The mean score for Responses was significantly higher than the mean score for Underlying Causes (*t*(210) = 4.839, Cohen’s *d* = 0.333). Finally, the mean score for the Personal Support ARTIC 45 subscale was significantly higher than the mean score for System Support (*t*(118) = 10.641, Cohen’s *d* = 0.975). Results are summarized in Table 3.

**Table 3:** Summary statistics and t test results for ARTIC 35, ARTIC 45, and subscale scores

Variable	M	SD	Min	Max	*n*
ARTIC 35 Overall	5.149	0.665	3.171	6.886	211
On-the-Job Behavior	5.416	0.690	3.286	7.000	211
Self-Efficacy	5.328	0.952	2.286	7.000	211
Reactions	5.200	0.816	2.857	7.000	211
Responses	5.014	0.902	2.286	7.000	211
Underlying Causes	4.787	0.793	1.857	7.000	211
ARTIC 45 Overall	5.081	0.514	3.867	6.356	119
Personal Support	5.045	1.050	2.200	6.800	119
System Support	3.498	0.700	2.200	7.000	119
Paired Comparisons	***M*** **Diff**	***SE Diff***	***t***	***p***	***Cohen’s d***
Job Behavior vs. Underlying Causes	0.630	0.050	12.597	< 0.001*	0.867
Job Behavior vs. Responses	0.402	0.047	8.859	< 0.001*	0.591
Job Behavior vs. Reactions	0.217	0.048	4.551	< 0.001*	0.313
Job Behavior vs. Self-Efficacy	0.088	0.056	1.563	0.120	0.108
Self-Efficacy vs. Underlying Causes	0.542	0.062	8.736	< 0.001*	0.601
Self-Efficacy vs. Responses	0.314	0.065	4.863	< 0.001*	0.335
Self-Efficacy vs. Reactions	0.129	0.058	2.207	0.028	0.152
Reactions vs. Underlying Causes	0.413	0.055	7.457	< 0.001*	0.513
Reactions vs. Responses	0.186	0.059	3.135	0.002*	0.216
Responses vs. Underlying Causes	0.228	0.047	4.839	< 0.001*	0.333
Personal Support vs. System Support	1.547	0.145	10.641	< 0.001*	0.975

*p <.003(Bonferroni adjusted level of significance)

### Research Question Two: TIC Training Versus no Training

The second research question examined differences in ARTIC scores between those who received prior training or education in TIC and those who did not. Prior TIC training may have been received from YMHFA or another source. Mean scores for the overall ARTIC 35 and its five subscales were compared using an independent *t* test assuming equal variances. The mean score for the overall ARTIC 35 was higher for trained participants (*M* = 5.306, *SD* = 0.631) than untrained participants (*M* = 4.947, *SD* = 0.657), a significant difference (*t*(209) = 4.027, *p* <.001, Cohen’s *d* = 0.642). The mean score for On-the-Job Behavior was higher for trained participants (*M* = 5.553, *SD* = 0.633) than untrained participants (*M* = 5.239, *SD* = 0.723), a significant difference (*t*(209) = 3.361, *p* <.001, Cohen’s *d* = 0.674). The mean score for Self-Efficacy was higher for trained participants (*M* = 5.502, *SD* = 0.887) than untrained participants (*M* = 5.104, *SD* = 0.991), a significant difference (*t*(209) = 3.070, *p* =.001, Cohen’s *d* = 0.933). The mean score for Reactions was higher for trained participants (*M* = 5.354, *SD* = 0.817) than untrained participants (*M* = 5.000, *SD* = 0.775), a significant difference (*t*(209) = 3.193, *p* <.001, Cohen’s *d* = 0.799). The mean score for Responses was higher for trained participants (*M* = 5.168, *SD* = 0.817) than for untrained participants (*M* = 4.815, *SD* = 0.971), a significant difference (*t*(209) = 2.865, *p* =.001, Cohen’s *d* = 0.887). Finally, the mean score for Underlying Causes was higher for trained participants (*M* = 4.951, *SD* = 0.814) than untrained participants (*M* = 4.575, *SD* = 0.716), a significant difference (*t*(209) = 3.508, *p* <.001, Cohen’s *d* = 0.773). Results are summarized in Table 4.

**Table 4:** Comparison of means for ARTIC 35 subscales for TIC training versus no TIC training

	Untrained		Trained		Comparison Test		
ARTIC Score	M	SD	M	SD	t (209)	*p*	Cohen’s d
Overall (35)	4.947	0.657	5.306	0.631	4.027	< 0.001*	0.642
Job Behavior	5.239	0.723	5.553	0.633	3.361	< 0.001*	0.674
Self-Efficacy	5.104	0.991	5.502	0.887	3.070	0.001*	0.933
Reactions	5.000	0.775	5.354	0.817	3.193	< 0.001*	0.799
Responses	4.815	0.971	5.168	0.817	2.865	0.001*	0.887
Underlying Causes	4.575	0.716	4.951	0.814	3.508	< 0.001*	0.773

Trained: *N* = 119; Untrained: *N* = 92. *p <.003 (Bonferroni adjusted level of significance)

### Research Question Three: TIC Differences Across School Professional Roles

The third research question examined whether the cognitive, behavioral, and environmental attitudes related to TIC differed across school professional roles. Differences in mean scores for the ARTIC 35 and its subscales were compared using a one-way ANOVA, followed by Bonferroni post-hoc pairwise comparisons. Results indicated that there were significant differences in mean scores across the five categorical professional roles for overall ARTIC 35 (*F*(4, 206) = 4.367, *p* =.002, $\:{\eta\:}^{2}$= 0.078), Responses (*F*(4, 206) = 4.717, *p* =.001, $\:{\eta\:}^{2}$= 0.084), Underlying Causes (*F*(4, 206) = 3.938, *p* =.004, $\:{\eta\:}^{2}$= 0.071), Reactions (*F*(4, 206) = 2.673, *p* =.033, $\:{\eta\:}^{2}$= 0.049), and Self-Efficacy (*F*(4, 206) = 2.432, *p* =.049, $\:{\eta\:}^{2}$= 0.045), but not On-the-Job Behavior (*F*(4, 206) = 2.291, *p* =.061, $\:{\eta\:}^{2}$= 0.043). Results also indicated that there were no significant differences in mean scores across the five professional roles for overall ARTIC 45 (*F*(4, 114) = 1.494, *p* =.209, $\:{\eta\:}^{2}$= 0.050), nor was there significance for Personal Support (*F*(4, 114) = 1.445, *p* =.224, $\:{\eta\:}^{2}$= 0.048) or System Support (*F*(4, 114) = 1.217, *p* =.308, $\:{\eta\:}^{2}$= 0.041).

Bonferroni pairwise comparisons indicated the mean overall ARTIC 35 score for Counselors or Nurses (*M* = 5.520, *SD* = 0.561, *n* = 38) was higher than the mean for Teachers (*M* = 5.052, *SD* = 0.649, *n* = 145). The mean subscale for Responses was higher for Counselors or Nurses (*M* = 5.462, *SD* = 0.810, *n* = 38) than the mean for Teachers (*M* = 4.930, *SD* = 0.904, *n* = 145) and paraprofessionals (*M* = 4.507, *SD* = 0.462, *n* = 11). The mean subscale for Underlying Causes was higher for Counselors or Nurses (*M* = 5.147, *SD* = 0.742, *n* = 38) than the mean for Teachers (*M* = 4.697, *SD* = 0.779, *n* = 145) and paraprofessionals (*M* = 4.531, *SD* = 0.480, *n* = 11). The mean subscale for Reactions was higher for Counselors or Nurses (*M* = 5.568, *SD* = 0.673, *n* = 38) than the mean for Teachers (*M* = 5.093, *SD* = 0.771, *n* = 145). Finally, the mean subscale for Self-Efficacy was higher for Counselors or Nurses (*M* = 5.733, *SD* = 0.753, *n* = 38) than the mean for Teachers (*M* = 5.207, *SD* = 0.970, *n* = 145). Pairwise comparisons were not examined for On-the-Job Behavior, overall ARTIC 45, Personal Support, nor System Support due to non-significance in these one-way ANOVAs. Results are summarized in Table 5, where means without an overlapping subscript differed at the 0.05 level of significance in the Bonferroni pairwise comparisons.

**Table 5:**
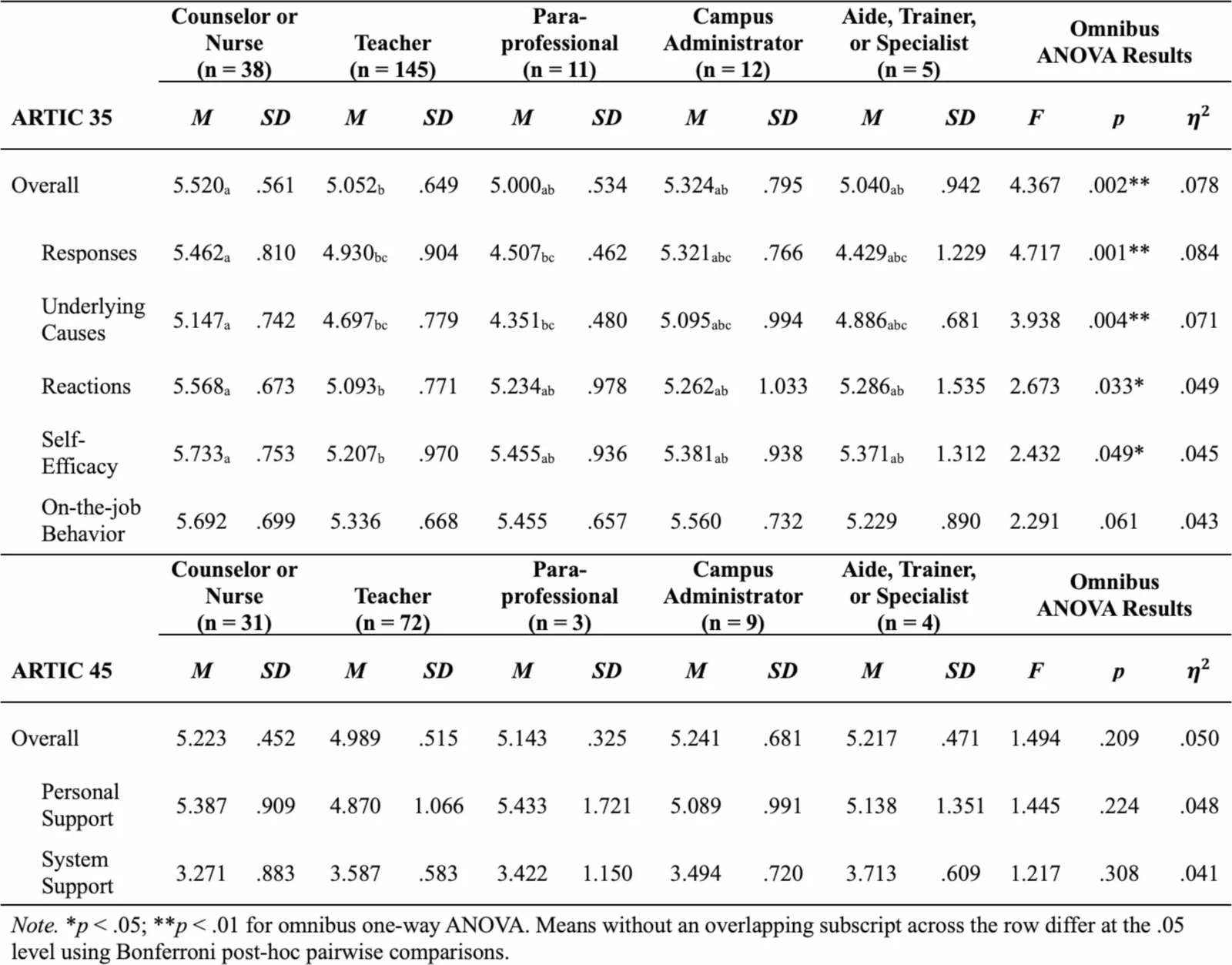
Results from the one-way ANOVA and pairwise comparisons across professional roles

## Discussion

A compelling finding in this study’s demographics is the number of responses per campus to the question “*What is your primary campus?*” The answer options for this question included elementary (i.e., PreK-5th grade), middle (i.e., 6th −8th grade), and high school (i.e., 9th − 12th grade). Almost 50% of the responses were from elementary school campuses (*n* = 102), 17% were from middle school campuses (*n* = 35), and 35% were from high school campuses (*n*= 74). It raises the question of why so many elementary school professionals were interested in participating in this study and what compelled them to do so, compared with middle and high school professionals. Anecdotally, often, behavioral issues are not equated with elementary school children, with a mindset that they are too young to exhibit symptoms and behavioral problems associated with trauma. However, this demographic finding could speak to the growing concern about youth in the U.S. exhibiting an earlier onset of trauma symptoms and behaviors, with more intensity and prevalence occurring in the school setting, especially since the COVID-19 pandemic (Geiser 2019; Hertz and Barrios 2021) U.S. Department of Education, 2021. This finding is further substantiated by a comment made by the sample school district’s Deputy Superintendent about teachers and school professionals raising verbal concerns about the increased incidence of behavioral issues occurring among elementary school children in the classroom, some of which could be described as aggressive (Deputy Superintendent, personal communication, May 16, 2025). Although the data collected in this study do not include a qualitative rationale for why school professionals chose to participate, it stands to reason that elementary school professionals may have done so out of concern and heightened awareness that a problem exists.

The TIC attitude scores in some subscales of the ARTIC were significantly higher than others for the overall mean across subscales, and for the paired *t* test comparisons. Ideally, there should be no significant differences among subscale scores because, theoretically, attitude scores should be equally high across all subscales. Of the five core ARTIC scales examined in this study, the On-the-Job behavior subscale was significantly higher for the overall ARTIC, as well as the paired *t*test comparisons. Followed by the Self-Efficacy subscale, which also scored significantly higher on the overall ARTIC, as well as the paired comparisons, than the other ARTIC subscales. The paired relationship between the On-the-Job and Self-Efficacy subscales was not significant, indicating that attitudes on both subscales were high (i.e., favorable toward TIC). The On-the-Job behavior subscale endorses empathy-focused staff behavior versus control-focused staff behavior, and measures how school professionals believe they behave in real-time situations with students who have experienced trauma. The Self-Efficacy subscale endorses feeling capable of meeting the demands of working with a traumatized population versus feeling unable to do so (Baker et al. 2021). These findings suggest that the attitudes of the school professionals in this sample are confident and committed in their capability to apply TIC, remain regulated, calm, and supportive during student escalations, collaborate effectively with colleagues and families, and manage emotionally taxing aspects of trauma at work. Differences between paired comparisons of the Reactions, Responses, and Underlying Causes subscales were also significant but had lower mean differences. This suggests that some school professionals in the sample view reactions to the work, responses to the problem, and the underlying causes of problem behavior as intentional and fixed, resulting in a choice to ignore trauma behaviors as opposed to problem behaviors being viewed as malleable, flexible, and encouraging seeking support. Overall, the findings associated with the*t* test analysis could be used to design a training program, workshop, or educational intervention explicitly tailored for the needs of school professionals using a top-down or bottom-up approach based on scale outcomes. For example, a top-down approach for the school professionals in this sample would be tailored from the highest to the lowest scale scores (i.e., On-the-Job, Self-Efficacy, Reactions, Responses, and Underlying Causes). In this case, interventions would be tailored with the understanding that school professionals are already confident in their ability and skills to apply TIC in real-time situations, and more educational emphasis would be placed on concepts associated with Reactions, Responses, and Underlying Causes of behavior.

Attitudes related to TIC are significantly higher for those who have received TIC training versus those who have not for all five of the core ARTIC subscales. Likewise, (Cohen 1988)guidelines for interpreting effect sizes were used to determine the magnitude of the difference, which indicates large effect sizes (> 0.8) for all core ARTIC subscales. Based on these findings, TIC training is fostering favorable attitudes toward TIC among school professionals in this school district, indicating that the training is working. Additionally, exposure to the YMHFA training that the district is implementing may represent an important step toward organizational readiness by enhancing knowledge, confidence, and supportive attitudes consistent with trauma-informed principles. This corroborates findings from previous studies and programs demonstrating that TIC training is effective in improving TIC attitudes (MacLochlainn et al. 2022) U.S. Department of Education,^2021^. Given the significance of these study findings, a next step to consider might be to model future efforts after The Purple Lanyard Project—an initiative designed to raise awareness of and support for youth mental health, especially in schools (U.S. Department of Education, 2021. The idea is that wearing a purple lanyard symbolizes that a person has completed YMHFA training and is a trusted adult available for support. Likewise, incorporating students into the mental health training could be equally beneficial. For example, the Teen Mental Health First Aid Training (tMHFA) is also evidence-based and trains teens to help their peers who may be experiencing emotional distress, a mental health crisis, or substance abuse (National Council for Mental Wellbeing, 2026. Training everyone in the school district and the broader school community about TIC offers wide-reaching and transformative benefits by creating a healthier, more empathetic, and more effective learning environment. Moreover, comprehensive, system-wide training remains essential for sustainable organizational transformation. These findings suggest that even modest exposure to trauma-related education and training may positively influence attitudes toward implementation readiness.

Study findings in the TIC-trained group for the ARTIC 45, which contains the two supplemental subscales, were significant in the *t* test comparison for the Personal Support and System Support subscales. This suggests that, in this sample, school professionals were supportive and confident in their individual implementation of TIC, but may not feel supported by colleagues or administrators in implementing TIC. Several leading organizations in TIC strongly advocate for a system-wide approach in which trauma-informed practices are embedded across entire organizations or school systems, rather than at the individual level (NCSEA, 2019; NEA, 2025; SAMHSA, 2023; National Child Traumatic Stress Network, 2017. That said, these study findings are not surprising given the recent national push for schools to become trauma-informed, which has left many schools in the U.S. lagging as they struggle to fully embrace or implement TIC/TIAs due to various systemic barriers.

Analysis of the one-way ANOVA suggests that attitudes toward TIC across school roles indicate that counselors and nurses have significantly more favorable attitudes than teachers on most subscales, except for the On-the-Job behavior subscale. This indicates that all school roles in this sample have favorable attitudes about their ability to apply TIC at work, under pressure in real-time situations. This aligns with findings in the *t-*test comparisons examined in research question one, where we found that mean scores for the On-the-Job behavior subscale were significantly higher than all other ARTIC subscales. It is not surprising that counselors and nurses would have more favorable attitudes toward TIC since their educational preparation includes TIC concepts and practices, which is further supported by organizations that govern the practice of school nursing and school counseling (American Association of Colleges of Nursing, 2025; American School Counselor Association, 2025; National Association of School Nurses, 2021. Conversely, while not universally mandated, some states do incorporate TIC education into their teacher education curricula/programs (i.e., California, Washington, and Massachusetts). However, this is an emerging trend that is not yet widespread, making TIC content an important component of teacher professional development post-certification (American Association of Colleges for Teacher Education, 2025.

Additionally, teachers are undoubtedly the first point of contact for students, as they spend the most time with them daily, making their buy-in to TIC strategies and principles crucial. A key to earning teacher buy-in centers on motivation for classroom use, which comes from understanding relevance and feasibility and offering support. Trust-Based Relational Intervention (TBRI) is an established trauma-based framework that can be adapted for use in school settings (Purvis et al., 2013). Teachers, as well as the school-wide community, would benefit greatly from TBRI training, which focuses on connecting, empowering, and correcting strategies for use in the classroom. Emerging evidence supports the implementation of TBRI in schools by school professionals, resulting in improved student behavior and the support of trauma-informed practices (Parris et al. 2015; Reid 2018; Stipp and Kilpatrick 2021). Likewise, integrating TBRI with instructional coaching with teachers where TBRI-trained staff, such as counselors, school nurses, or TBRI practitioners, work alongside teachers to model trauma-informed behaviors, provide support, and feedback could be powerful, especially when paired with an implementation science framework such as the Consolidated Framework for Implementation Research (Damschroder et al. 2022). Although SAMHSA’s TIC framework does not specifically endorse any intervention models such as TBRI, the TBRI principles align closely with SAMHSA’s broader TIC concepts and framework. The SAMHSA framework defines TIC, while TBRI provides the intervention.

What is interesting is the low response rate to the ARTIC survey among school nurses (*n* = 1) compared with school counselors (*n* = 37). Research shows the critical role that school nurses play in the delicate balance between student health, well-being, and learning, yet they are often excluded from academic leadership teams and conversations (CDC 2024a, ; (Willgerodt et al. 2018). This can result in school nurses’ perception that they are not part of the academic team despite their evidence-based value in facilitating educational success. (Maughan et al. 2018) found that school nurses want greater collaboration with teachers and counselors, especially around student mental and behavioral health needs. However, in some schools and districts, insufficient administrative support often presents an obstacle for school nurses.

### Limitations

Although study outcomes provide an informed baseline for the school district’s readiness for a trauma-focused educational intervention (i.e., SAMHSA’s second step in becoming trauma-informed), several study limitations must be considered. First, there is limited generalizability due to an 8.5% response rate, which can be considered low. Response rates to online surveys in educational settings vary considerably, with large ranges of what may be considered acceptable. Online surveys commonly yield response rates between 10% and 30%, with some education-specific studies reporting an average of between 28 and 44%, depending on several factors such as the recruitment and sampling strategy, and administration method used (National Survey of Student Engagement, 2021; Wu et al., 2022).

Second, there is a risk of nonresponse bias due to the large number of school professionals who began the survey but did not complete it. In the current study, the initial response rate prior to data cleaning was 14.1% (*N* = 348), which falls within an acceptable range for online surveys. During data cleaning, it was noted that several respondents started the survey but, for whatever reason, did not finish and were therefore removed from the final data set for non-completion, resulting in an 8.5% response rate (*N =* 211). Anecdotally, partial survey completion may be attributable to several factors, including time constraints, conflicting interests, competing demands, technical challenges, and unfamiliarity or discomfort with TIC concepts.

Third, the lower response rate raises the possibility of engagement bias, whereby school professionals with greater interest in or exposure to TIC are more likely to complete the survey. This parallels the concept of superutilizers in health services research, which refers to individuals who engage with health systems due to greater need or relevance (Soril et al. 2015). Hence, study outcomes might not be representative of the full sample. Likewise, engagement bias might also explain why some school professionals started the survey but did not complete it.

Fourth, differences in some aspects of the demographic make-up for trained and untrained participants may confound comparisons across these two groups. Ideally, the groups being compared would be similar in most aspects except having TIC training, but these demographic differences in race/ethnicity, highest level of education, primary campus, and primary role limit these comparisons. This indicates that differences in attitudes might be due to individual differences versus differences in TIC training.

Finally, the purpose of the study was to establish a baseline of the school district’s readiness to implement TIC strategies and approaches. As such, prior exposure to trauma-related training, regardless of format, has the potential to influence readiness and was not limited to YMHFA training alone. Accordingly, this study did not differentiate between participants who had received YMHFA training and those who had not, as some participants may have completed alternative TIC-related training prior to the implementation of Texas-mandated mental health training. Therefore, on the ARTIC survey, when participants were asked to self-report whether they had received prior TIC training, it could have been through YMHFA or another TIC training program. Using self-report introduces the possibility of self-report bias, as responses were based on participants’ perceptions and recall, which may lead to variability in how they interpreted what qualified as prior TIC training.

Given the study’s limitations, the findings should be interpreted with caution and warrant further exploration by the research team to better understand the underlying factors influencing participant responses and to enhance the rigor and generalizability of future research.

## Implications and Future Research

Outcomes from the current study provide critical baseline data to inform organizational readiness for change aimed at improving school professionals’ attitudes and confidence related to TIC, offering a foundation for tailoring future interventions within the district. These results can be used to identify strengths and gaps across roles, facilitating targeted professional development. Likewise, findings from this study can help other school districts that seek trauma-informed initiatives by providing a practical template for assessing organizational readiness. The quantitative approach used in this study offers a replicable method for districts to evaluate their own baseline using validated instruments. By adapting this approach to their local context, districts can generate their own data to guide strategic planning and support the phased implementation of TIC practices. Establishing a data-driven baseline is a critical first step in fostering sustainable, system-wide trauma-informed change.

Although considerable discussion has focused on implementing TIC approaches in schools, relatively few studies have demonstrated complete alignment with the SAMHSA guidelines for organizational transformation (MacLochlainn et al. 2022). Many school-based TIC initiatives focus narrowly on training or classroom strategies without addressing broader systems change at the district level, thereby limiting their scope. The application of implementation science, a field focused on understanding how to adopt and integrate evidence-based practices into real-world settings, is essential for ensuring a successful transition from knowledge translation to practice (Avery et al. 2021). Future research would benefit from using a framework to guide implementation strategies, such as the Consolidated Framework for Implementation Research (CFIR) (Damschroder et al. 2022). A second study, using the same school district as the current research, is in the design phase and plans to use a qualitative approach to identify barriers and facilitators to implementing TIC in the district. Having the quantitative data from the current study, coupled with the qualitative data to be gathered in the upcoming follow-up study, provides a more comprehensive and well-rounded organizational assessment. This mixed-method approach aligns with best practices in implementation science and lays a strong foundation for initiating step two of SAMHSA’s (2023) guide: building organizational capacity for implementation.

The state of mental health in the U.S. is widely regarded as being in crisis. One of the most significant challenges facing the U.S. government is the urgent need to incorporate more TIC policies at the state level, rather than just a few. This need is driven by the consistently high prevalence of mental illness among youth and adults (National Alliance on Mental Health, 2024, which calls for a coordinated response that begins with implementing policies at the state level that have a trickle-down effect on public organizations such as schools (Marris 2024; Uglean 2024). However, the lack of consistency among states about passing TIC policies has left disparities in how U.S. schools respond to trauma and mental health needs. Several states do not have any TIC policies (i.e., Idaho, Alabama, and Iowa). Some states have passed only one policy (i.e., North Dakota, South Dakota, Alaska, Wyoming, and Ohio). Twenty states have more than six trauma-informed policies (Marris 2024)p. 4). Some states, like Texas, have made considerable progress toward legislative changes to support their residents’ mental well-being. Evidence of change is evident in the newly instituted legislative requirement in Texas that all school professionals receive mental health training (TEA House Bill 3, 2023). While Texas is one of the few states leading the effort to make TIC training a priority, one area where Texas lags is in requiring trauma or ACEs screening in schools (Marris 2024; Uglean 2024). The Texas Education Code § 38.351 (2024) prohibits the screening of any student as part of the process to determine a need for early intervention for mental health or substance abuse. In contrast, empirical research demonstrates that structured mental health screening programs in U.S. schools can significantly improve early identification and intervention, leading to improved long-term outcomes for students (Husky et al. 2011; Levitt et al. 2018). Future research to consider includes piloting a program that incorporates voluntary, universal trauma and/or ACEs screening for all students, much like schools already screen for other issues that affect health and learning.

The SCT served as a useful framework for conceptualizing trauma-informed attitudes; however, this study’s purpose was not to test the theory or theoretical relationships. Future research should investigate the extent to which the SCT constructs explain variation in trauma-informed attitudes and organizational readiness among school employees. Such work may contribute to theory-driven models to support system-wide implementation of TIC in schools.

## Conclusion

Study findings suggest that the sample school district demonstrates a strong level of confidence in its ability to apply TIC practice, particularly in real-time, on-the-job situations. This readiness provides a solid foundation for organizational transformation. The SAMHSA guide (2023) provides a research-backed, structured roadmap for schools to adopt trauma-informed approaches. Following the SAMHSA implementation guide will allow the district to move beyond individual training efforts toward a comprehensive, system-wide approach grounded in evidence-based principles. Findings from this study offer valuable insight for tailoring explicit future interventions that align with the unique needs of the sample district, fulfilling the core objective of step one in SAMHSA’s trauma-informed implementation framework: a baseline organizational assessment.

## Data Availability

The author(s) confirm that all data generated and analyzed during this study are included in the article and are available upon request.

## Declarations

### Artificial Intelligence

The authors did not use any Large Language Models in the study conception, design, data collection, analysis, findings, writing, or editing of this manuscript.
